# Bone Grafting

**Published:** 1891-02

**Authors:** 


### Bone Grafting.

In *The Lancet* is reported a case of bone grafting with decalcified bone chips at the Edinburgh Royal Infirmary. The case was that of a farmer, aged forty-four, with chronic inflammation of the bursa under the right ligamentum patellæ, and abscess in the head of the tibia. The swelling was opened and the gelatinous contents removed, the abscess cavity cleaned out, a drainage tube introduced, the wound dressed antiseptically, and a splint applied. The tibial cavity did not show signs of closing, so that it was determined to stuff it with decalcified bone. A piece of ox's rib was scraped, decalcified with weak hydrochloric acid, pressed clean, soaked in carbolic solution—one in twenty—for forty-eight hours, and cut in small pieces. The tibia was scraped and gouged to afford free access to the cavity, which was stuffed with the decalcified bone shavings. As these did not entirely fill the cavity the operator added the pieces of bone that had been removed and kept in boric acid solution. The cavity was two inches in diameter. It closed very rapidly, but the probe could still enter about an inch, and bare bone could be felt. Several pieces of necrotic bone were removed, being evidently those that had been gouged out and returned at the time of the operation. A very small sinus remained when the patient was discharged, but was soon reported to be closed. Dr. Miller remarks that the closure of the cavity was facilitated by the use of decalcified bone, as recommended by Senn, and that this was superior to the fresh bone, which was actually a hindrance. The bone cavity quickly filled with granulations, but the external wound remained open till all the necrosed bits of fresh bone came away. There was never a drop of pus, and the temperature never rose over 100° F. The dressing employed was iodoform and wood wool mostly, and all dressing was done under the spray.—*Times and Register.*

THE subject of asepsis and antisepsis is again brought to mind by the free discussion in the late congress. The whole subject resolves itself into one word, according to our mind: "Cleanliness," aided or unaided by antiseptic agents.

